# Gestures speed up responses to questions

**DOI:** 10.1080/23273798.2024.2314021

**Published:** 2024-02-17

**Authors:** Marlijn ter Bekke, Linda Drijvers, Judith Holler

**Affiliations:** a Donders Institute for Brain, Cognition and Behaviour, Radboud University, Nijmegen, the Netherlands; b Max Planck Institute for Psycholinguistics, Nijmegen, the Netherlands

**Keywords:** Multimodal language, gesture, turn-taking, conversation, facilitation, response times

## Abstract

Most language use occurs in face-to-face conversation, which involves rapid turn-taking. Seeing communicative bodily signals in addition to hearing speech may facilitate such fast responding. We tested whether this holds for co-speech hand gestures by investigating whether these gestures speed up button press responses to questions. Sixty native speakers of Dutch viewed videos in which an actress asked yes/no-questions, either with or without a corresponding iconic hand gesture. Participants answered the questions as quickly and accurately as possible via button press. Gestures did not impact response accuracy, but crucially, gestures sped up responses, suggesting that response planning may be finished earlier when gestures are seen. How much gestures sped up responses was not related to their timing in the question or their timing with respect to the corresponding information in speech. Overall, these results are in line with the idea that multimodality may facilitate fast responding during face-to-face conversation.

## Introduction

In face-to-face conversations, people rapidly take turns at talking: responses to questions typically occur within 0-200 ms after question end (Stivers et al., 2009). Slower response times can mark responses as dispreferred (Kendrick & Torreira, 2015; Schegloff, 2007) or indicate weaker social connection (Templeton et al., 2022). Thus, response times in conversation matter, and therefore fast language processing and response planning are of the essence.

During conversational turn-taking people also use bodily signals to communicate (Bavelas, 2022; Kendon, 2004). These signals may facilitate fast language processing (Drijvers & Holler, 2022; Holler & Levinson, 2019) and thus allow for earlier responses. We tested this hypothesis by investigating whether iconic co-speech hand gestures speed up responses to questions.

Iconic hand gestures depict semantic information about concrete referents like actions or objects (McNeill, 1992), e.g. depicting drinking by bringing a hand to the mouth, as if holding a glass. These gestures could facilitate fast responding in several (not mutually exclusive) ways. Responding in conversation requires comprehension of the incoming utterance and simultaneous response planning, after which the response is launched when turn-final cues are detected (Bögels et al., 2015; Levinson, 2016; Levinson & Torreira, 2015). Gestures could facilitate comprehension and response planning by, for example, adding semantic information that helps grasp the utterance’s meaning (Dargue et al., 2019; Holler et al., 2018), increasing attention to speech (Dargue et al., 2019; Holler et al., 2018), or improving prediction of upcoming words (Holler & Levinson, 2019; ter Bekke et al., 2024; Zhang et al., 2021). Subsequently, gestures could result in responses being *launched* earlier by gesture retractions signalling upcoming turn ends (Duncan, 1972; Holler et al., 2018; Levinson & Torreira, 2015). Using an experimental button press paradigm which encouraged participants to respond as fast as possible (irrespective of whether the speaker had finished speaking), we focused on the first stage – the possibility that iconic gestures influence when participants *can* respond (i.e. gestures facilitating response planning).

Gestures may possibly not speed up or even slow down response planning. Parallel comprehension and response planning is cognitively taxing. If listeners additionally have to process visual signals and integrate them with speech where relevant, this may significantly strain our processing system and slow down responses (Holler & Levinson, 2019).

Correlational corpus data favour accounts postulating a facilitative role of visual signals: questions with hand gestures get faster responses than questions without (Holler et al., 2018; ter Bekke et al., 2024). However, no causal claims can be made based on corpus data, making it unclear if gestures speed up responses, or a confounding variable causes the effect. For example, questions people gesture with might generally be easier to understand or structured differently, enabling earlier responses.

Controlled experimental studies also reported gestural speed up effects. When participants saw videos with speech and iconic gestures (versus speech-only), they were faster to judge whether the videos matched actions (Kelly et al., 2010) or pictures (Krason et al., 2021; Wu & Coulson, 2015), or whether they felt addressed by the speaker (Nagels et al., 2015). These results align with lower-level multimodal facilitation effects where participants are faster to detect audiovisual stimuli versus audio-only (e.g. Miller, 1986).

However, gestural speed up effects were not always found. Participants did not read words aloud faster when preceded by videos with a pantomime of the word (Bernardis et al., 2008). Similarly, participants did not judge faster whether someone described an object- or person-related event when they also produced emblems or pantomimes (He et al., 2015). In three other studies (Drijvers et al., 2018; Drijvers et al., 2019; Drijvers et al., 2019), seeing an actress produce iconic gestures did not speed up word recognition.

Altogether, the experimental evidence that gestures speed up responses is mixed. Although these studies differed in several ways, their tasks involved participants making judgements (e.g. about videos matching pictures), rather than planning responses that are contingent on previous utterances as in conversation. This makes it difficult to directly link these results to the corpus finding that questions with gestures got faster responses.

Our key aim was to test the gestural speed up effect experimentally, to bring the corpus and experimental findings together. Participants viewed videos in which an actress asked them yes/no-questions (e.g. “is being able to type well useful if you’re a secretary?”) and had to answer as fast and accurately as possible, via button press. The exact same questions were presented either with or without a corresponding iconic gesture (e.g. fingers pretending to type). We opted for responding via button press rather than verbally for several reasons. Firstly, this way we retained control over the complexity and duration of the responses, which are known to affect their timing (Roberts et al., 2015). Secondly, buttons can be easily pressed *during* a question without the norms and principles governing overlap management in conversation coming into play (Sacks et al., 1974). This gives us a more direct measure of when recipients *can* respond (the focus on the present study), rather than when recipients *would* respond in more conversational settings.

If gestures speed up responses, this would support the idea that multimodal language is easier/faster to process than unimodal language (Drijvers & Holler, 2022; Holler & Levinson, 2019). It would advance the literature by showing that iconic co-speech gestures specifically are one of the signals contributing to multimodal facilitation.

Moreover, visual signals produced earlier in the question may exert greater influence on fast responding (for such effects of eyebrow movements, see Nota et al., 2022). As the gesture timing spontaneously varied across questions (i.e. one gesture may occur earlier than another), we explored whether gestures especially sped up responses when they had earlier preparations, strokes or retractions (Kita et al., 1998), which refer to the hand(s) moving from their resting position into gesture space and preparing the gestural handshape (preparation phase), depicting the action or object (stroke phase), and returning to the resting position (retraction phase). Furthermore, if gestures speed up responses by improving predictions about upcoming words, then gestures that precede their corresponding word in speech more (i.e. more predictive potential) may speed up responses more (ter Bekke et al., 2024).

## Methods

The pre-registration is available here: https://aspredicted.org/dp573.pdf.

Data, scripts and supplementary materials can be found here: https://osf.io/e6wpt/.

### Participants

Sixty right-handed native speakers of Dutch (51 women, *M*_age_ = 23;2 [years;months], *SD *= 4;6) participated. All reported normal hearing, (corrected-to-)normal vision, and no neurological/motoric/language-related disorders (same for pre-test participants). Participants gave informed consent before the experiment and received financial compensation/course credits. The experiment was approved by the Social Sciences Faculty Ethics Committee, Radboud University (approval code: ECSW-2018-135).

### Stimuli

Participants watched short videos of a native Dutch actress asking easy yes/no-questions. Questions were factual to ensure 50% yes-responses and 50% no-responses. Personal questions were included as fillers. For a list of all questions, see https://osf.io/e6wpt/.

**Figure 1.**
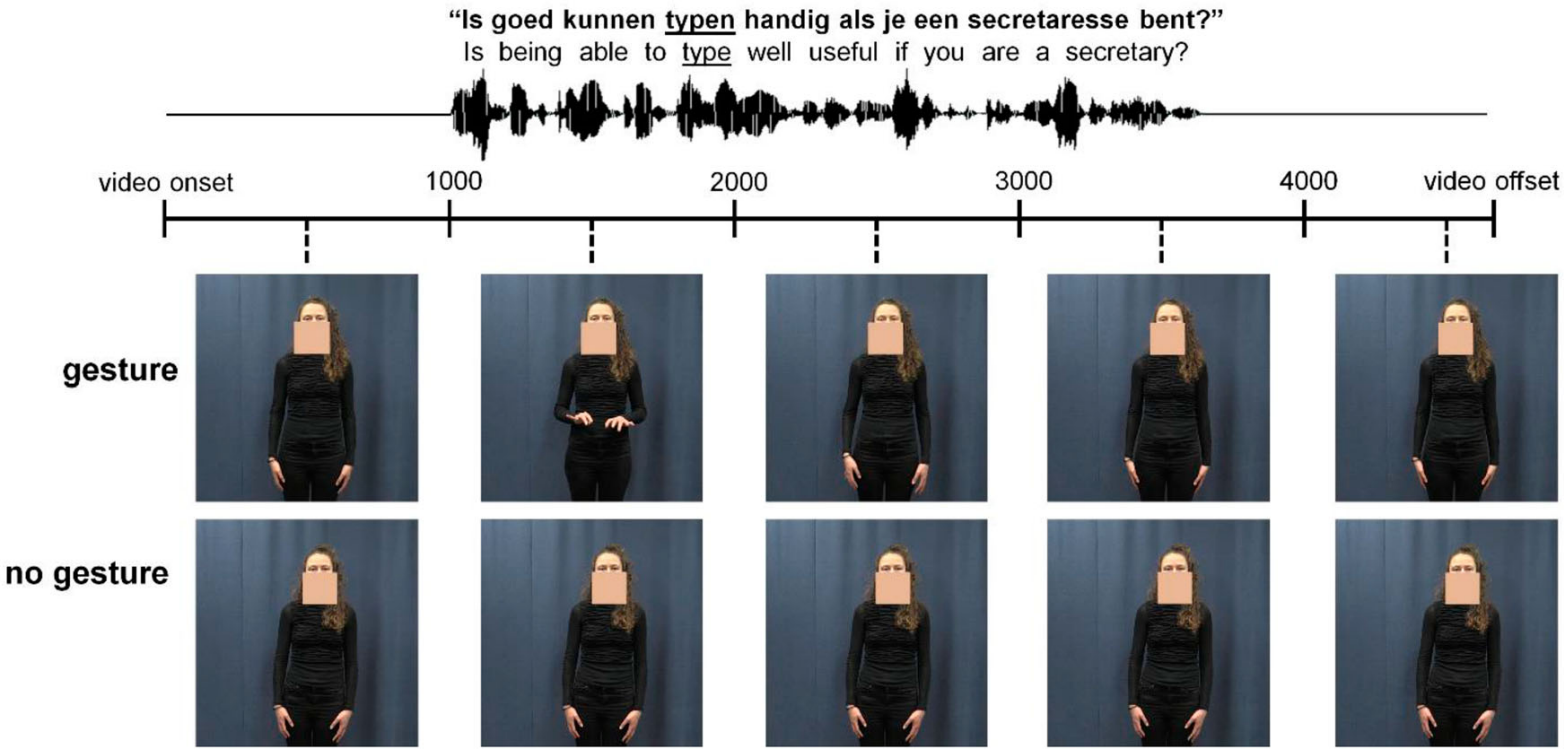
Schematic stimulus overview.

Videos were recorded with a Canon XF205 camera (50fps) and displayed the actress from head to knees (Figure 1). She was instructed to utter each question as she normally would. In the Gesture condition, she was asked to produce a gesture depicting the lexical affiliate (i.e. the word in the question corresponding to the gesture) in a way that felt natural. All gestures were iconic depictions (e.g. pretending to swim for “swimming”), except one which was therefore excluded, reducing the number of items. A pre-test with 20 different participants confirmed the gestures (without audio) in general fit the lexical affiliates (see Appendix A). Twelve gestures (7 fillers) were not well-understood and excluded.

To test how well, in their question context, the gestures fit their lexical affiliates (iconicity), and how natural they looked, a second pre-test showed the gesture clips *with* audio to another 20 different participants (see Appendix B). Gestures generally fit the lexical affiliates (mean iconicity rating 5.0 on 1–7 scale, range: 3.1 - 6.8) and were rated as rather natural (mean naturalness rating 4.8 on 1–7 scale, range: 3.2 - 6.2). As these ratings varied considerably across items, we explored whether they predicted how much each gesture sped up responses.

A skin-coloured square was added in Adobe Premiere Pro to cover visible speech movements from the face/throat (Figure 1). This was done for both conditions, allowing us to use the Gesture condition audio also in the No-gesture condition. Audio was intensity-scaled (70 dB) and denoised in Praat (Boersma, 2001). Because gestures could start before or end after the question, videos were cut from 1000 ms before to 1000 ms after the question. On average, gestures started 188 ms before question onset (*SD *= 311 ms) and ended 999 ms before question offset (*SD *= 616 ms).

### Coding

We annotated gesture phases frame-by-frame in ELAN (version 5.5; Wittenburg et al., 2006). Based on the hands being blurry or clear, gestures were segmented into static or dynamic movement phases (Seyfeddinipur, 2006) and identified as preparation, stroke, retraction, or pre- or post-stroke hold (i.e. hands are still before or after the stroke; Kita et al., 1998). Gesture phase coding was reliable (Appendix C).

### Procedure

In this online study (Gorilla; Anwyl-Irvine et al., 2020), participants were instructed to participate in a quiet location, in one go, with wired ear-/headphones and other computer programs closed. A questionnaire ensured eligibility. After a sound check, the main experiment started. Participants were instructed to watch each video and answer the question as quickly and accurately as possible via button press, without waiting until question end. They were not informed about the gesture manipulation.

Each trial started with a 1000 ms fixation cross, followed by the video. Participants responded with a *yes* or *no* button press (button assignment was counterbalanced), after which a new trial started immediately.

After three practice trials per condition, the stimuli were presented in four blocks with self-paced breaks in between. Each participant saw 134 items^1^ (32 fillers) in a within-subjects design: 67 in each condition (16 fillers). Within each condition, the amount of yes- and no-responses was counterbalanced. Across participants each item was seen in both conditions (50% Gesture; 50% No-gesture). Stimulus order was pseudo-randomized per participant, with no more than two repetitions of one condition in a row. Finally, participants were debriefed.^2^

### Analysis

We excluded five responses slower than 5000 ms (0.1% of data; three in the Gesture condition), eight responses before question onset (0.1%; four Gesture), and three cases where the video did not play (one Gesture). For response time analyses, we excluded 276 responses with incorrect answers (4.5%; 129 Gesture).

We fitted (generalised) linear-mixed effects models using lme4 (1.1-26; Bates et al., 2015) in R (4.2.1; R Core Team, 2019) and lmerTest (3.1-1; Kuznetsova et al., 2017). Factors were sum-to-zero contrast coded (−0.5, 0.5). Continuous predictors causing scaling errors were z-standardized. We used maximal random effects structures. In case of convergence issues, we used estimates from the non-converged fit as starting values, and then compared the estimates from different optimisers using allFit(). If these converged to highly similar values, the warnings were considered false positives (RDocumentation, 2021). This occurred for all analyses below.

To test whether Condition (Gesture, No-gesture) affected accuracy (0, 1) and response times (in ms), we used models with Condition as predictor. To test whether gestures with earlier gesture phases (preparation, stroke, retraction) especially sped up responses, we calculated the difference between each gesture phase onset and question offset (in ms), and used this as predictor. This slightly deviates from our pre-registration. First, we tested the effect of Gesture phase onset in interaction with Condition, not as main effect, because gesture phase onset timing should only matter in the Gesture condition (where participants actually saw this gesture phase). Second, the preparation and stroke onset analyses were added. To explore whether gestures that preceded their lexical affiliate more sped up responses more, we calculated the difference between the preparation/stroke onset and the lexical affiliate onset and used this difference as a predictor of response times.

To explore whether iconicity and naturalness scores predicted how much gestures sped up responses, we used a data-driven model-building procedure, starting with only Condition as predictor and adding interactions stepwise (Iconicity*Condition first). We only kept factors/interactions if they significantly improved model fit.

## Results

### Accuracy

**Figure 2.**
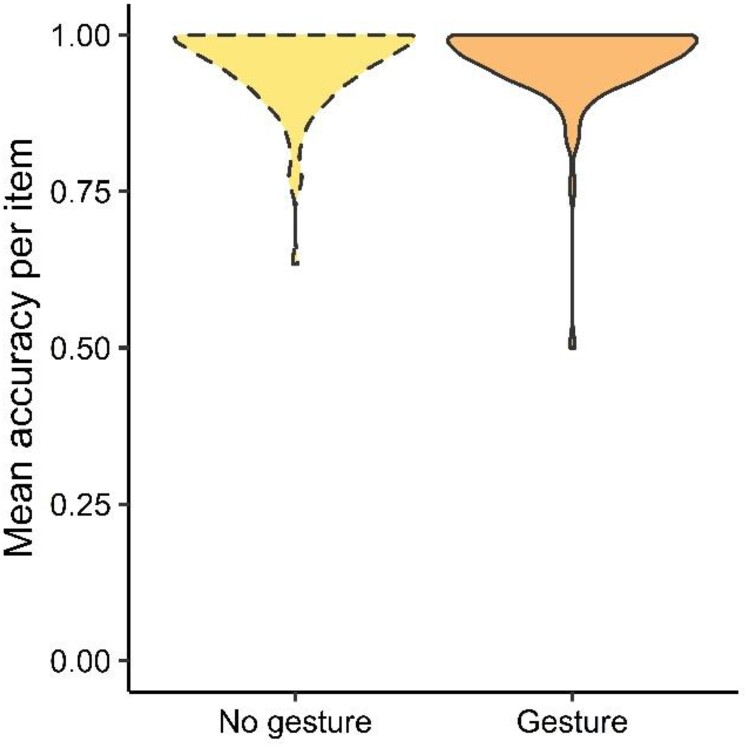
Accuracy did not differ by condition. Distributions of mean accuracy for each item are shown per condition.

The questions were easy to answer, with 95.5% (*SD = *20.8%) correct responses (Gesture: 95.8%, *SD* = 20.1%, No-gesture: 95.2%, *SD* = 21.4%) (Figure 2). Accuracy did not differ between questions with and without gestures (*β* = 0.25, *SE* = 0.19, *z* = 1.29, *p *= 0.20).

### Response times

**Figure 3.**
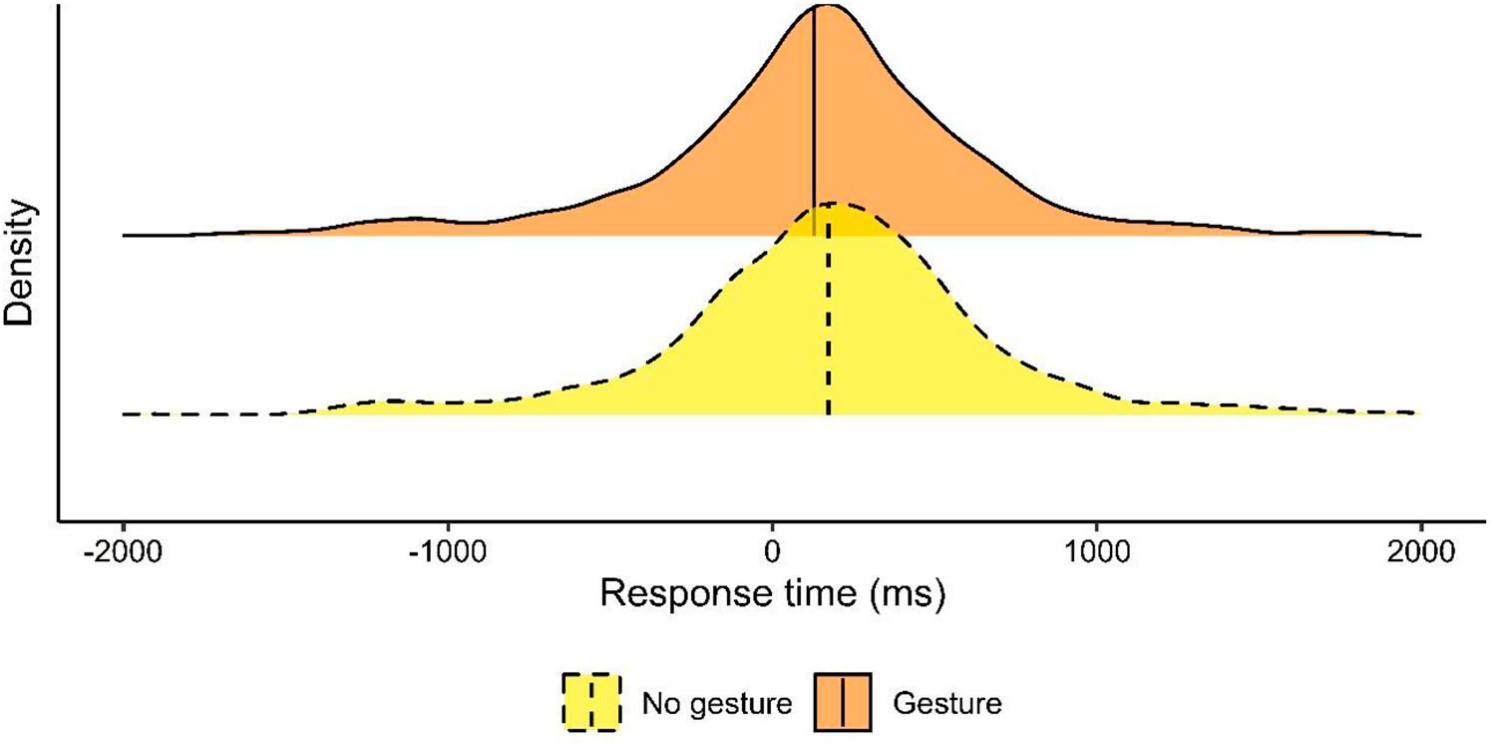
Gestures sped up responses to questions. The response time distribution per condition is shown. Vertical lines display mean response times.

Overall, the mean response time was 160.6 ms (*SD* = 529.94 ms). Without gestures, it was 186.9 ms (*SD* = 524.2 ms) and with gestures around 50 ms faster (134.5 ms, *SD* = 534.4 ms; Figure 3). Indeed, gestures sped up responses to questions^3^ (*β* = −52.65, *SE* = 10.81, *t* = −4.87, *p *< 0.01).

### Gesture phase timing

How early gesture phases started in the question did not predict how much gestures sped up responses, for preparations (*β* = 12.29, *SE* = 10.92, *t* = 1.13, *p *= 0.26), strokes (*β* = 11.96, *SE* = 10.87, *t* = 1.10, *p *= 0.27), nor retractions (*β* = 14.30, *SE* = 10.96, *t* = 1.31, *p *= 0.20).

### Predictive potential

All preparations started before their lexical affiliate, on average 753 ms before (*SD *= 268 ms). Moreover, most strokes (81%) started before their lexical affiliate, on average 227 ms before (*SD *= 225 ms). How much preparations (*β* = 0.05, *SE* = 0.04, *t* = 1.35, *p *= 0.18) or strokes (*β* = 0.06, *SE* = 0.05, *t* = 1.43, *p *= 0.15) preceded lexical affiliates did not predict how much gestures sped up responses.

### Gesture iconicity and naturalness

Iconicity (*β* = 2.87, *SE* = 11.98, *t* = 0.24, *p *= 0.81) and naturalness ratings (*β* = 0.19, *SE* = 16.24, *t* = 0.01, *p *= 0.99) did not predict how much gestures sped up responses.^4^

## Discussion

In face-to-face conversation, people rapidly take turns and use hand gestures to communicate. This study provides the first experimental evidence that these gestures speed up responses to questions, in line with corpus work showing that questions with gestures get faster responses (Holler et al., 2018; ter Bekke et al., 2024) and the *multimodal facilitation hypothesis* stating that communicative bodily signals facilitate fast language processing (Drijvers & Holler, 2022; Holler & Levinson, 2019). Despite limited cognitive capacities and strong time pressure (in conversation, and likewise in our experiment), seeing iconic hand gestures facilitates fast responding.

Using an experimental button press paradigm which encouraged participants to respond as fast as possible, we focused on whether iconic gestures influence when participants *can* respond (i.e. gestures facilitating response planning), rather than when participants would respond in actual conversation, where turn-taking principles and conversational pragmatics influence response timing (Kendrick & Torreira, 2015; Sacks et al., 1974). The present study showed that the perception of gestures led to faster responses. Critically, the perception of gestures did not lead to responses being more closely timed to the question end (see Supplementary Materials) and gestures did not speed up responses more if retractions (a potential turn-final cue; Duncan, 1972). We thus interpret the results as suggesting that gestures allow for faster response planning, possibly due to faster comprehension. To address the different question of whether gestures may influence when participants *launch* their responses in actual conversation, as gestures can also function as turn-final cues and manage turn transitions in conversation (Duncan, 1972; Holler et al., 2018; Kendrick et al., 2023; Mondada, 2006; Zellers et al., 2019), a paradigm able to simulate the sensitivities surrounding gaps and overlaps in turn-taking would be needed.

The mechanism underlying this gestural facilitation may be gestures providing semantic information (Dargue et al., 2019; Dargue & Sweller, 2018; Holler et al., 2018) or increasing attention to speech (Dargue et al., 2019; Holler et al., 2018). In this study, it appears unlikely that gestures sped up responses by facilitating prediction of upcoming words (Holler & Levinson, 2019; ter Bekke et al., 2024; Zhang et al., 2021). Although most gestures started before their lexical affiliates, similar to natural conversation (Donnellan et al., 2022; ter Bekke et al., 2020; ter Bekke et al., 2024), gestures did not speed up responses more when gestures occurred earlier. However, as gesture timing was not experimentally manipulated, we cannot draw strong conclusions here. Future studies may delve deeper into the underlying mechanisms of gestural facilitation, for example by varying the task or stimuli (e.g. additional control conditions to contrast possible mechanisms), or by looking at individual differences and how these relate to the underlying mechanisms (Özer & Göksun, 2020).

Interestingly, our gestural speed up effect was smaller (∼50 ms faster) than in past corpus studies (∼300 ms faster). Perhaps gestures are more effective in an interactive, social context. Thus, our experimental task, with video stimuli rather than a live interlocutor, and button press responses rather than verbalised responses, may have reduced gestures’ effect. Alternatively, it is possible that the effect size attributed to gestures in the corpus studies was partly caused by confounding variables. Many factors influence response times (e.g. question duration; Roberts et al., 2015), and one of these may have correlated with gesture presence. This highlights the importance of complementing corpus studies with controlled experiments that use identical questions with and without gesture, but also the need for building on the present findings with more ecologically valid, interactionally-embedded experimental paradigms. Such studies could, for example, manipulate gesture presence in the context of other cues (e.g. visible speech, gaze), let participants respond verbally, or add interactivity or co-presence. Overall, converging evidence from approaches varying from more ecologically valid to more experimentally controlled seems best suited to uncover robust patterns in multimodal language processing.

In conclusion, we demonstrated that iconic gestures speed up responses to questions. While previously found in corpora, our experimental evidence is the first to showing iconic gestures’ causal role in fast responding. Despite the challenge of processing and integrating different signals under significant time constraints, multimodality may actually facilitate fast responding.

## Supplementary Material

Supplemental Material

## Notes

1 Two items were excluded to ensure proper counterbalancing.

2 Participants’ Empathy Quotients were also collected but did not associate with how much participants benefited from seeing the gesture. For these supplementary analyses, see https://osf.io/e6wpt/.

3 Gestures did not make response times more precise (i.e. closer to question end), but rather sped up responses in general, making overlap responses less precise and gap responses more precise on average. For these supplementary analyses, see https://osf.io/e6wpt/.

4 Iconicity also did not modulate the relation between gesture phase timing (with respect to question offset or lexical affiliate onset) and how much gestures sped up responses. For these supplementary analyses, see https://osf.io/e6wpt/.
