# Supplementary material for: Gestures speed up responses to questions
Source: Lang Cogn Neurosci. 2024 Feb 17;39(4):423–30. doi: 10.1080/23273798.2024.2314021 (PMC11132552; doi:10.1080/23273798.2024.2314021)
Supplement: Supplemental Material [file PLCP_A_2314021_SM4830.zip › Appendices.docx]

Appendices

Appendix A: Pre-test 1 (without audio)

A pre-test confirmed that the gestures fit the lexical affiliates. Twenty right-handed native Dutch speakers (13 women, seven men, Mage = 25;11 [years;months], SD = 5;3) participated. In this online experiment (Qualtrics, Provo, UT), participants watched 148 silent Gesture videos and typed in three guesses about what each gesture depicted. After guessing, the lexical affiliate was shown and participants indicated how well they thought it fit the hand movement on a Likert scale of one (not at all) to seven (very well). The lexical affiliate or a synonym was typed in on the first guess for 29.7% of the gestures, and on all three guesses together for 37.2%, showing that gestures can be ambiguous in the absence of speech (Hadar & Pinchas-Zamir, 2004). The mean Likert score was 5.0 (this word fits the hand movement), indicating that in general our lexical affiliates fit the gestures.

If an item had a mean three-guess recognition score below 15% and a mean Likert score below four (n = 28), we decided on a qualitative, case-by-case basis whether to exclude it. We considered that in the main experiment gestures would be seen in the question context, which is known to improve comprehension (Hadar & Pinchas-Zamir, 2004; Kelly et al., 2010). If the guesses showed participants almost grasped the meaning, we included it. Twelve items were excluded (7 fillers).

Appendix B: Pre-test 2 (with audio)

To test how fitting and natural the final gestures were in their question context, in a second pre-test the Gesture clips were shown with audio. Twenty right-handed native Dutch speakers (19 women, one man, Mage = 24;8 [years;months], SD = 6;2) participated who had not done the first pre-test. After watching each clip, participants indicated whether the gesture fit the lexical affiliate on a scale of one (not at all) to seven (very well) (iconicity ratings). Next, they indicated how natural they thought the gesture was in the question context on a scale of one (not at all natural) to seven (very natural) (naturalness ratings). The mean iconicity rating of 5.0 (range: 3.1 - 6.8) indicated that the gestures generally fit the lexical affiliates. The mean naturalness rating of 4.8 (range: 3.2 - 6.2) indicated that the gestures were generally more natural than unnatural.

Appendix C: Gesture coding

To obtain reliability of the gesture phase coding, we calculated modified Cohen’s kappa and raw agreement using EasyDIAg (Holle & Rein, 2015). This method is suitable for when coders need to create segmentations based on a continuous stream of video data, which they subsequently label. EasyDIAg first links annotations from coders together and then checks agreement. Annotations from coders were linked if they overlapped 60%, following Holle & Rein (2015). Average disagreement between main coder MtB and reliability coder LvO on preparation onset started was 11 ms (raw agreement 98.7%, modified Cohen’s kappa 0.97), based on 20.4% of the gestures (n = 21). For stroke onset average disagreement was 19 ms (raw agreement 98.7%, modified Cohen’s kappa 0.97) and for retraction onset this was 14 ms (raw agreement 98.7%, modified Cohen’s kappa 0.97). Cohen’s kappa values of 0.81-1.00 were interpreted as almost perfect agreement, following Landis & Koch (1977). In case of disagreement, coding from the main coder was used.
