# Supplementary material for: Gestures speed up responses to questions
Source: Lang Cogn Neurosci. 2024 Feb 17;39(4):423–30. doi: 10.1080/23273798.2024.2314021 (PMC11132552; doi:10.1080/23273798.2024.2314021)
Supplement: Supplemental Material [file PLCP_A_2314021_SM4830.zip › SupplementaryMaterials.docx]

**Supplementary analyses**

***Reaction time precision***

Overall, the mean response time precision (i.e. absolute value of response times; Corps et al., 2018) was 402.4 ms (*SD* = 380.4 ms). Without gestures, it was 405.2 ms (*SD* = 381.4 ms) and with gestures around 6 ms faster (399.5, *SD* = 379.5 ms; Figure SM1). A linear mixed effects model with log-transformed Response time precision (ms) as outcome variable, Condition as predictor and maximal random effects structure confirmed that gestures did not lead to responses being timed more precisely to question end (*β* = -0.05, *SE* = 0.03, *t* = -1.37, *p* = 0.18).


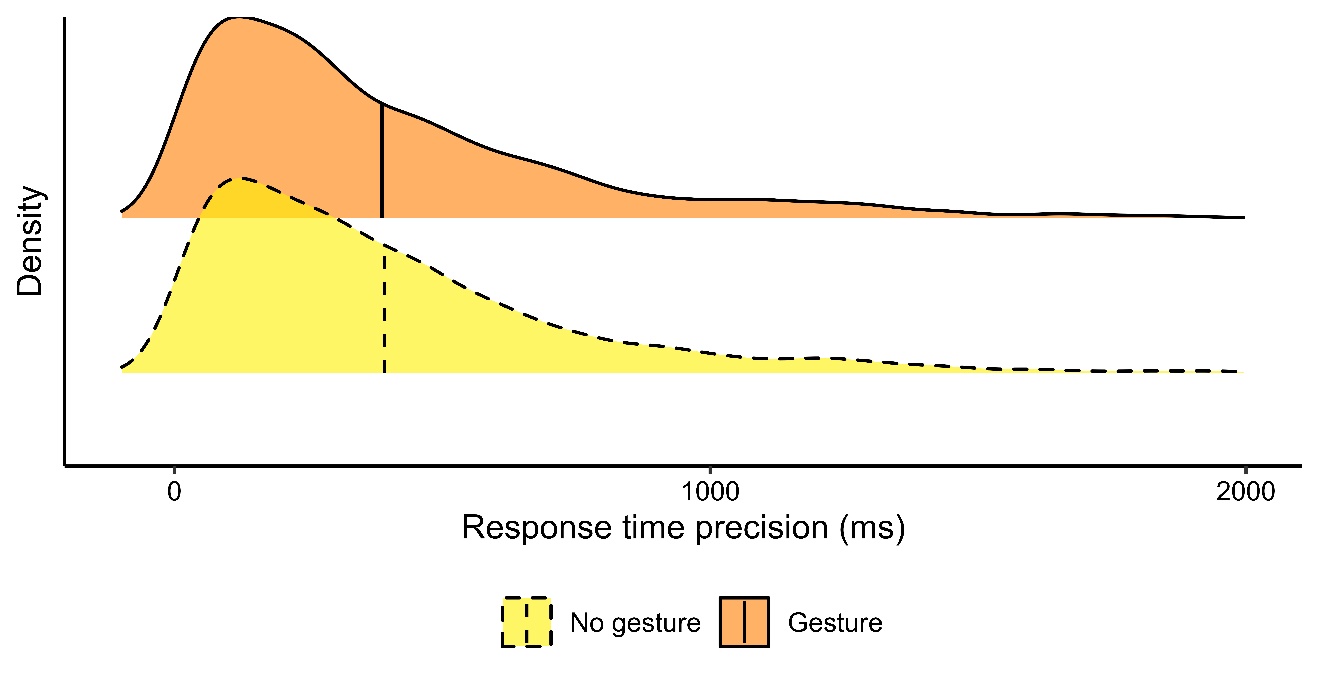


*Figure SM1.* Reaction times were similarly precise in both conditions. The distribution of reaction time precision per condition is shown. Vertical lines display mean reaction time precision.

***Gaps and overlaps***

Although the participant responses are a continuous distribution and express the same phenomenon, for analytic purposes, we next split up the responses into overlap responses (i.e. responses occurring before question end; response time < 0) and gap responses (i.e. responses occurring after question end; response time > 0). In the Gesture condition, 32.4% of responses occurred in overlap, and in the No-gesture condition 30.8% of responses occurred in overlap. The response times per condition separately for gaps and overlaps show that participants responded faster in the Gesture condition both when the responses were issued in overlap with the question (Gesture: *M* = -409 ms, *SD* = 410 ms; No-gesture: *M* = -355 ms, *SD* = 349 ms) and when they were issued after the question had ended (Gesture: *M* = 395 ms, *SD* = 364 ms; No-gesture: *M* = 428 ms, *SD* = 349 ms; Figure SM2)^[[1]](#footnote-1)^. In sum, gestures sped up responses overall, irrespective of when the responses occurred.


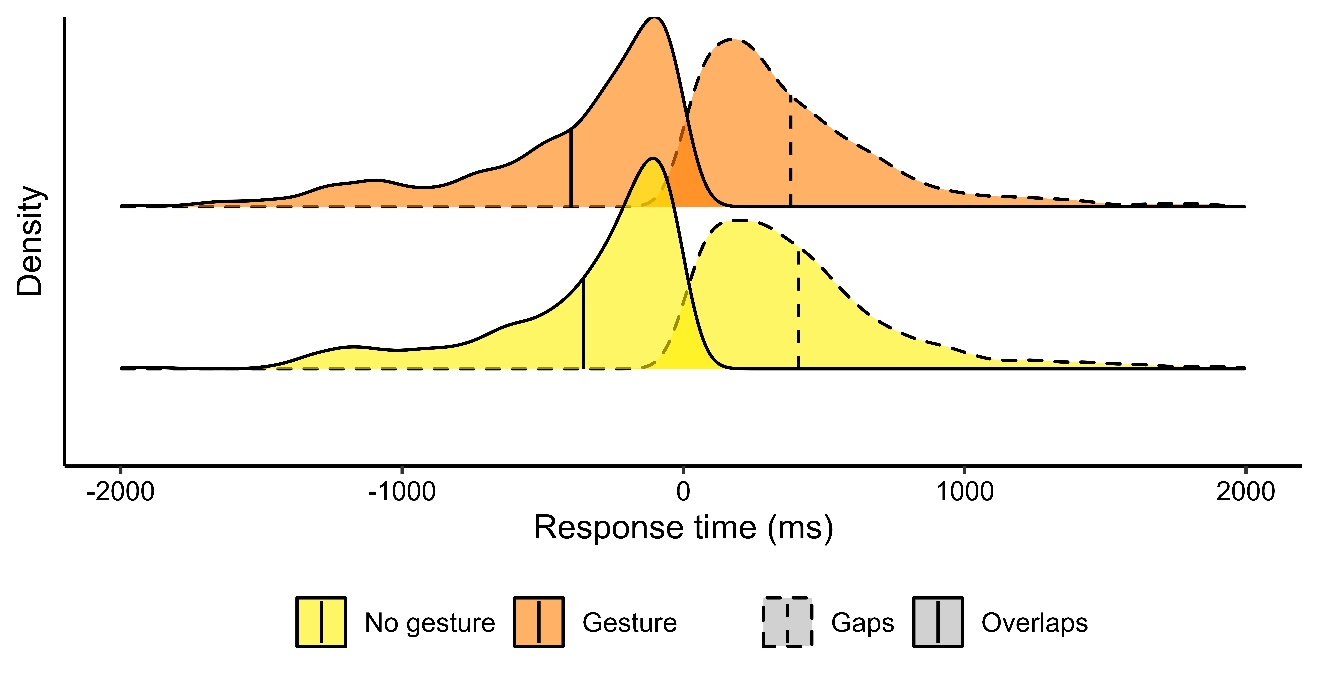


*Figure SM2.* Gestures sped up reaction times both for responses issued in overlap with the question and after question end. The distribution of reaction times per condition split by response type (issued in overlap with the question (overlaps) or after question end (gaps)) is shown. Vertical lines display mean reaction times.

***Individual differences: Empathy Quotient***

Participants’ Empathy Quotients did not predict how much gestures sped up their responses (*β* = 1.88, *SE* = 1.35, *t* = 1.40, *p* = 0.17).

***Gesture phase timing and iconicity***

The relation between gesture phase timing with respect to question offset and the extent to which gestures sped up response times did not depend gesture iconicity, for preparations (*β* = -15.00, *SE* = 12.91, *t* = -1.16, *p* = 0.25), nor strokes (*β* = -16.77, *SE* = 12.65, *t* = -1.33, *p* = 0.19).

***Predictive potential and iconicity***

The relation between gesture phase timing with respect to lexical affiliate onset and the extent to which gestures sped up response times did not depend gesture iconicity, for preparations (*β* = 12.04, *SE* = 14.30, *t* = -0.84, *p* = 0.40), nor strokes (*β* = 14.03, *SE* = 14.04, *t* = 1.00, *p* = 0.32).

1. Note that the category of responses issued after question ends (gaps) for the Gesture condition may consist of different items than the category of responses issued after question ends for the No gesture condition. This makes it harder to directly compare the conditions. Because of this, and because splitting the data into responses issued after question ends (gaps) and during questions (overlaps) creates smaller datasets, we did not statistically test whether Condition impacts reaction times separately for each of these categories (gaps and overlaps). [↑](#footnote-ref-1)
